# Rejuvenation in invertebrates and their relevance for ageing biology

**DOI:** 10.1098/rstb.2026.0005

**Published:** 2026-11-12

**Authors:** Mickaël Wurtz, Aldine R. Amiel, Eric Röttinger

**Affiliations:** Institute for Research on Cancer & Aging, Nice (IRCAN), Université Côte d'Azur, CNRS, INSERM, Nice, France

**Keywords:** rejuvenation, nonvertebrates, ageing reversal, lifespan, cnidarians

## Abstract

Ageing is not merely the progressive and irreversible accumulation of damage and a decline in biological function; it may also reflect the persistent and progressive dysregulation of developmental mechanisms during adulthood, leading to reduced stress resilience. This perspective has stimulated interest in rejuvenation, defined as the partial reversal of biological age, for example, through experimental reprogramming approaches such as heterochronic parabiosis or Yamanaka factor-based reprogramming. Although rejuvenation in mammals remains limited and requires strong experimental interventions, naturally occurring age-reversal phenomena have been described in several invertebrate metazoans, including ctenophores, cnidarians, platyhelminths, annelids and nematodes. This has been described either at the morphological level or at the physiological, cellular and/or molecular levels. Here, we review and synthesize studies documenting these inductive processes, with particular attention to terminology and to the distinction between ‘morphological reversion’ and multi-level ‘rejuvenation’ associated with potential lifespan extension. When available, we also summarize the associated molecular evidence and discuss how systematic and in-depth, multi-level studies of this naturally occurring phenomenon could provide new opportunities for ageing biology and rejuvenation strategies in mammals.

This article is part of the theme issue ‘Developmental circuits in ageing’.

## 1. Introduction

Ageing has long been considered an irreversible, progressive loss of physiological integrity that humankind has attempted to prevent or revert at multiple times. The myth of the fountain of youth, the elixir of eternal youth, and even the alchemists’ philosopher's stone are myths that represent the desire of humankind to remain young forever, or even to turn back the ageing clock.

However, the first scientific step toward rejuvenation, i.e. turning back the ageing clock, was made only during the 19th century by Paul Bert, who performed the first recorded parabiosis experiment. He surgically joined two rats at their flanks and showed that the animals shared their bloodstream [1]. While it was initially conducted to study skin transplantation, this experiment led subsequently to heterochronic (young to old individuals) parabiosis that revealed longevity benefits and restored cell and tissue functions for older individuals [2–4]. Recent studies have revealed numerous improvements at the physiological and molecular levels, e.g. cognition or muscle strength for the old individual, via specific molecular signals such as GDF11 ([5], reviewed in [6]).

Twenty years ago, the discovery of four Yamanaka factors—OCT3/4, SOX2, KLF4 and MYC—enabled cell reprogramming [7]. While the expression of these four factors (4F reprogramming) enabled the de-differentiation of cells into pluripotent states, it was also observed that the cells can be rejuvenated by resetting the epigenetic and transcriptomic landscape towards younger/embryonic states [8,9]. Recent studies have shown that partial 4F reprogramming, which maintains original cell identity, still reverses ageing markers, including improved stress resilience [10]. At the whole-organism level, using cyclic 4F reprogramming in progeria mouse models, Ocampo and colleagues reduced the hallmarks of ageing (e.g. DNA damage) and extended the median lifespan of the mice by approximately 30% ([11], reviewed in [12]).

Thus, heterochronic parabiosis and 4F reprogramming are two very active and promising methods for reversing ageing markers in mammals, expanding their healthspan and, ultimately, lifespan. Nonetheless, various therapeutic challenges remain to avoid (i) uncontrolled cell dedifferentiation or (ii) organ-, tissue- or cell state-specific responses that may decrease efficiency or lead to the emergence of pathologies (e.g. teratomas and organ failure; [13], reviewed in [14]).

Interestingly, reversing the biological clock, following certain stimuli or spontaneously, is a property observed in some invertebrate metazoans, such as ctenophores, cnidarians, planarians, annelids and nematodes, since the 19th century (table 1, figure 1). These invertebrates have been described to undergo ‘reverse development’ (RD) or ‘rejuvenation’ and thus appear to reverse the classical developmental/ageing trajectory (see below; [15]). These processes have been identified using heterogeneous methodologies and at different biological levels, depending on their respective life cycles. Furthermore, a wide range of inconsistent terminology has been used to describe ‘reverse development’ and rejuvenation. For instance, in one of the most emblematic cases, the immortal jellyfish *Turritopsis dohrnii*, its ‘rejuvenation’ process [28,62] has been variously referred to as ‘ontogeny reversal’ [29], ‘reverse development’ [20] or even ‘life cycle reversion’ [63]. This terminological inconsistency, combined with the lack of a formal definition and standardized criteria to assess rejuvenation, complicates both comparisons across studies and conceptual clarity. In addition, a substantial proportion of described ‘rejuvenation’ occurrences relies only on morphological observations. While these observations provide a potential indication of rejuvenation, the absence of molecular and cellular evidence of reprogramming or rejuvenation raises important questions regarding the observed phenomenon, such as the following: what are those morphological changes related to? More specifically, do they really correspond to a rejuvenation process, or do they instead correspond to a stress-resistance stage or to another developmental trajectory? (see §7).

**Table 1.** Overview of metazoan species/phyla in which morphological reversion (MR) and/or rejuvenation have been described.

common name	phylum	(sub)-class or order	life cycle type	MR or rejuvenation sensitive life stage	reverted life stage	inductive stimuli	indicators of MR or rejuvenation				lifespan extension	sources
							morphology	physiology	cellular	molecular		
sea walnut	Ctenophora	Lobata	direct	mature lobate stage	cydippid stage	mechanical stress, starvation	yes	unknown	unknown	unknown	unknown	[15]
cauliflower coral	Cnidaria	Anthozoa	biphasic	primary polyp	planula-like	unfavourable conditions	yes	unknown	unknown	unknown	unknown	[16]
brown hydra	Cnidaria	Hydrozoa	direct	polyp	nd	asexual reproduction	unknown	yes	unknown	unknown	unknown	[17]
Christmas tree hydroid	Cnidaria	Hydrozoa	biphasic	polyp	nd	asexual reproduction	unknown	yes	unknown	unknown	unknown	[17]
hydrozoan jellyfish	Cnidaria	Hydrozoa	triphasic	early medusa buds	polyp	mechanical stress	yes	unknown	unknown	unknown	unknown	[18]
hydrozoan jellyfish	Cnidaria	Hydrozoa	triphasic	early medusa buds	polyp	mechanical stress	yes	unknown	unknown	unknown	unknown	[19]
smoothspined snailfur	Cnidaria	Hydrozoa	triphasic	early medusa buds	polyp	mechanical stress, caesium exposure	yes	unknown	unknown	unknown	unknown	[20,21]
marine hydrozoan	Cnidaria	Hydrozoa	unknown	polyp	planula	mechanical stress	yes	unknown	unknown	unknown	unknown	[22]
cytaeidid hydroid	Cnidaria	Hydrozoa	unknown	early medusa buds	stolon	unfavourable conditions	incomplete	unknown	unknown	unknown	unknown	[23]
hydrozoan	Cnidaria	Hydrozoa	triphasic	early medusa buds	stolon	unfavourable conditions	incomplete	unknown	unknown	unknown	unknown	[23]
clapper hydroid	Cnidaria	Hydrozoa	triphasic	early medusa buds	polyp	temperature shift	yes	unknown	unknown	unknown	unknown	[24]
hydrozoan jellyfish	Cnidaria	Hydrozoa	unknown	early medusa buds	polyp or planula-like	temperature shift	yes	unknown	unknown	unknown	unknown	[25]
sea fur	Cnidaria	Hydrozoa	triphasic	medusa stage	polyp	unknown	yes	unknown	unknown	unknown	unknown	[26]
hydrozoan	Cnidaria	Hydrozoa	triphasic	immature medusa	polyp	unknown	yes	unknown	unknown	unknown	unknown	[27]
immortal jellyfish	Cnidaria	Hydrozoa	triphasic	early and mature medusa	polyp	mechanical stress, starvation, temperature, salinity, caesium	yes	unknown	potentially	potentially	unknown	[20,28–32]
hydrozoan	Cnidaria	Hydrozoa	triphasic	early and mature medusa	polyp	mechanical stress	yes	unknown	unknown	unknown	unknown	[33]
hydrozoan	Cnidaria	Hydrozoa	triphasic	early and potentially mature medusa	polyp	mechanical stress	yes	unknown	unknown	unknown	unknown	[33–38]
crimson jelly	Cnidaria	Hydrozoa	triphasic	early and potentially mature medusa	polyp	mechanical stress	yes	unknown	unknown	unknown	unknown	[33–38]
moon jellyfish	Cnidaria	Scyphozoa	triphasic	polyp	pseudoplanula	mechanical stress	yes	unknown	unknown	unknown	unknown	[39]
moon jellyfish	Cnidaria	Scyphozoa	triphasic	ephyrae and mature medusa	polyp	mechanical stress, starvation, overcrowding	yes	unknown	unknown	unknown	unknown	[40]
crown jellyfish	Cnidaria	Scyphozoa	unknown	ephyrae	polyp	unfavourable conditions	yes	unknown	unknown	unknown	unknown	[41]
thimble jellyfish	Cnidaria	Scyphozoa	triphasic	ephyrae	polyp	unfavourable conditions	yes	unknown	unknown	unknown	unknown	[41]
crown jellyfish	cnidaria	Scyphozoa	triphasic	ephyrae	polyp	unfavourable conditions	yes	unknown	unknown	unknown	unknown	[41]
crown jellyfish	Cnidaria	Scyphozoa	triphasic	ephyrae	polyp	unfavourable conditions	yes	unknown	unknown	unknown	unknown	[41]
crown jellyfish	Cnidaria	Scyphozoa	triphasic	ephyrae	polyp	unfavourable conditions	yes	unknown	unknown	unknown	unknown	[41]
crown jellyfish	Cnidaria	Scyphozoa	triphasic	ephyrae	polyp	unfavourable conditions	yes	unknown	unknown	unknown	unknown	[41]
crown jellyfish	Cnidaria	Scyphozoa	triphasic	ephyrae	polyp	unfavourable conditions	yes	unknown	unknown	unknown	unknown	[41]
crown jellyfish	Cnidaria	Scyphozoa	triphasic	ephyrae	polyp	unfavourable conditions	yes	unknown	unknown	unknown	unknown	[41]
Mediterranean box jellyfish	Cnidaria	Cubozoa	triphasic	polyp to medusa metamorphosis	polyp	temperature shift	yes	unknown	unknown	unknown	unknown	[42]
marine flatworm	Platyhelminthes	Macrostomorpha	direct	mature adult	nd	mechanical stress	temporary	discussed	unknown	no	discussed	[43–47]
freshwater flatworm	Platyhelminthes	Tricladida	direct	mature adult	nd	mechanical stress	yes	yes	yes	yes	unknown	[48,49]
freshwater flatworm	Platyhelminthes	Tricladida	unknown	adult stage	nd	mechanical stress, starvation	unknown	yes	unknown	unknown	unknown	[17,50]
freshwater flatworm	Platyhelminthes	Tricladida	unknown	adult stage	nd	mechanical stress, starvation	unknown	yes	unknown	unknown	unknown	[17]
freshwater flatworm	Platyhelminthes	Tricladida	unknown	adult stage	nd	mechanical stress	unknown	yes	unknown	unknown	unknown	[17]
freshwater flatworm	Platyhelminthes	Catenulida	unknown	unknown	nd	asexual reproduction	unknown	yes	unknown	unknown	unknown	[17]
tapeworm	Platyhelminthes	Cyclophyllidea	triphasic	protoscolex and strobilated stage	hydatid cyst stage	unfavourable conditions	yes	unknown	unknown	unknown	unknown	[51]
tapeworm	Platyhelminthes	Cyclophyllidea	triphasic	protoscolex stage	hydatid cyst stage	unfavourable conditions	yes	unknown	unknown	unknown	unknown	[51]
tapeworm	Platyhelminthes	Cyclophyllidea	triphasic	protoscolex stage	hydatid cyst stage	unfavourable conditions	yes	unknown	unknown	unknown	unknown	[51]
marine bristle worm	Annelida	Eunicida	unclear (pot. biphasic)	adult stage	nd	asexual reproduction?, starvation	unknown	yes	unknown	unknown	unknown	(52)
aquatic oligochaete worm	Annelida	Tubificida	direct	immature adult stage	nd	mechanical stress	unknown	unknown	unknown	unknown	discussed	[53]
nematode	Nematoda	Rhabditida	biphasic	arrested-L1s, dauer stage	nd	exiting the diapause	no	yes	yes	yes	yes	[54–61]

**Figure 1.**
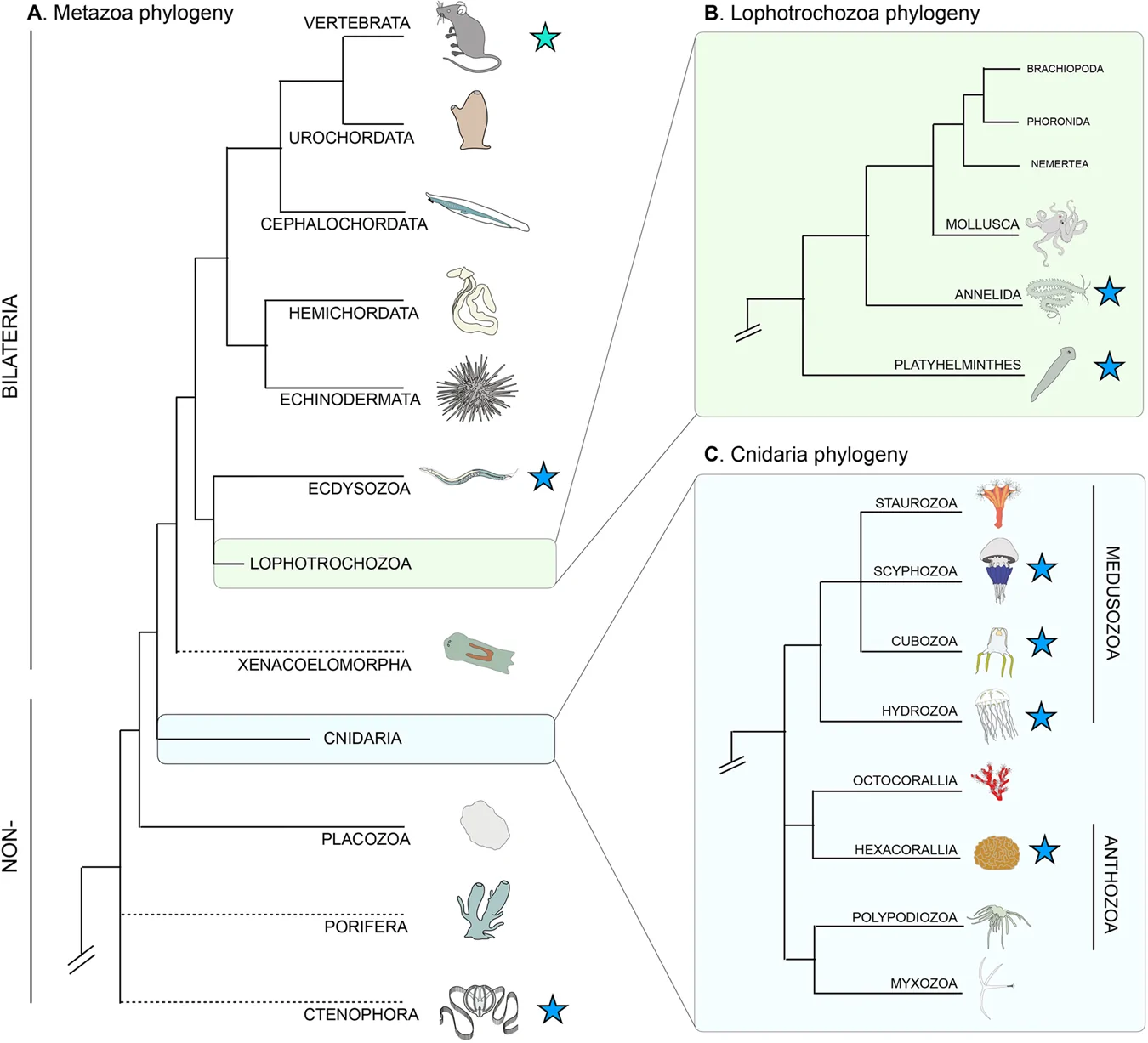
Simplified animal tree of life. (A) Metazoan phylogeny indicating the positions of the animal phyla in which morphological reversion and/or rejuvenation have occurred and that are addressed in this review: Ctenophora, Cnidaria, Lophotrochozoa and Ecdyzosoa. (B) Lophotrochozoan phylogeny highlighting two of the Lophotrochozoa phyla, Annelida and Platyhelminthes, in which morphological reversion and/or rejuvenation have been observed. (C) Cnidaria phylogeny highlighting four of the eight cnidarian phyla, Hydrozoa, Cubozoa, Scyphozoa and Hexacorallia, in which morphological reversion and/or rejuvenation has been observed. Stars indicate the phyla in which spontaneously/environmentally induced (blue) or experimentally induced (green*)* occurrences of this process have been reported. Dashed lines indicate positions that remain debated.

For the sake of clarity, here, we propose to distinguish between two definitions: *morphological reversion* (*MR*) and *rejuvenation*. MR can be defined as a return to a previously identified life stage on the basis of only the re-emergence of specific morphological criteria, whereas rejuvenation can be defined as the combination of the reversal of various characteristics, such as morphological, physiological, cellular and/or molecular traits, leading to the reversal of an organism's ageing trajectory. However, the presence of these features should not be interpreted as conferring equal weight in the assessment of rejuvenation, as their relevance may differ across biological levels and among hallmarks within each level. Using these definitions, we will review current knowledge on documented MR and rejuvenation in invertebrate metazoans and highlight recent advances in the field. We further propose complementary criteria to assess the impact of rejuvenation across diverse organisms and discuss how understanding the mechanisms driving rejuvenation in invertebrates might fuel mammalian rejuvenation and anti-ageing research. In this context, anti-ageing refers to interventions that slow, prevent or reverse aspects of biological ageing (molecular, cellular and physiological changes) and thereby extend healthspan, i.e. the number of years spent in good health, free from serious age-related disease.

This review is organized into five main sections, followed by a discussion and a conclusion. In the first section, we provide a non-exhaustive overview of the diverse life cycles observed in aquatic invertebrates. This provides background information for the documented MR (i.e. the return to a previous life stage) occurrences detailed in the second section. Here, we start by focusing on the most emblematic example, the immortal jellyfish *T. dohrnii*, which will be revisited on several occasions throughout the review. Additional examples of MR across diverse taxa have been organized according to their respective triggers rather than their taxonomic affiliation. This avoids redundancies and prevents a purely descriptive catalogue organization, allowing us to highlight common triggers underlying these processes. The third section addresses documented occurrences of rejuvenation in aquatic invertebrates. Subsequently, the fourth section addresses the rejuvenation processes identified in the nematode *Caenorhabditis elegans*. In the final section, we address a complementary aspect that has been documented but cannot be fully captured by the previous sections, i.e. the potential lifespan extension associated with MR/rejuvenation. This and other aspects (e.g. regeneration, asexual reproduction and rejuvenation) will be further addressed in the discussion section.

## 2. Life cycle complexity in aquatic invertebrates

To better understand the biological events relevant to the current topic, highlighted by various studies in aquatic invertebrate animals, we provide a brief non-exhaustive overview of their life cycle diversity. Aquatic animals can have complex life cycles that include a variety of life stages. While some can undergo *direct development* from the embryonic stage to the juvenile/adult stage (e.g. ctenophores and planarians [64,65], (figure 2A,B), others can undergo *indirect development* (e.g. cnidarians and annelids; figure 2C,D). This occurs either via a free-swimming larva that, in some species, can metamorphose into benthic (e.g. polyps, worms) or pelagic (e.g. medusae) life stages [66,67]. This is called a biphasic life cycle (figure 2C). In other cnidarian species, the free-swimming larva metamorphoses into an asexually reproducing benthic polyp that, in turn, can give rise to pelagic and sexually mature medusa by budding/strobilation [67]. This is called a triphasic life cycle (figure 2D). In some scyphozoan cnidarians (e.g.* Aurelia aurita*), the strobilating polyp (called scyphistoma) gives rise to a characteristic free-swimming ‘intermediate’ stage called the ephyra (an incomplete juvenile jellyfish) that progressively develops into an adult medusa [68] (figure 2D, light green). These examples illustrate not only the complexity of aquatic animal life cycles [69] but also their biological plasticity, which serves as a reference for the life cycle reversal events that have been reported thus far.

**Figure 2.**
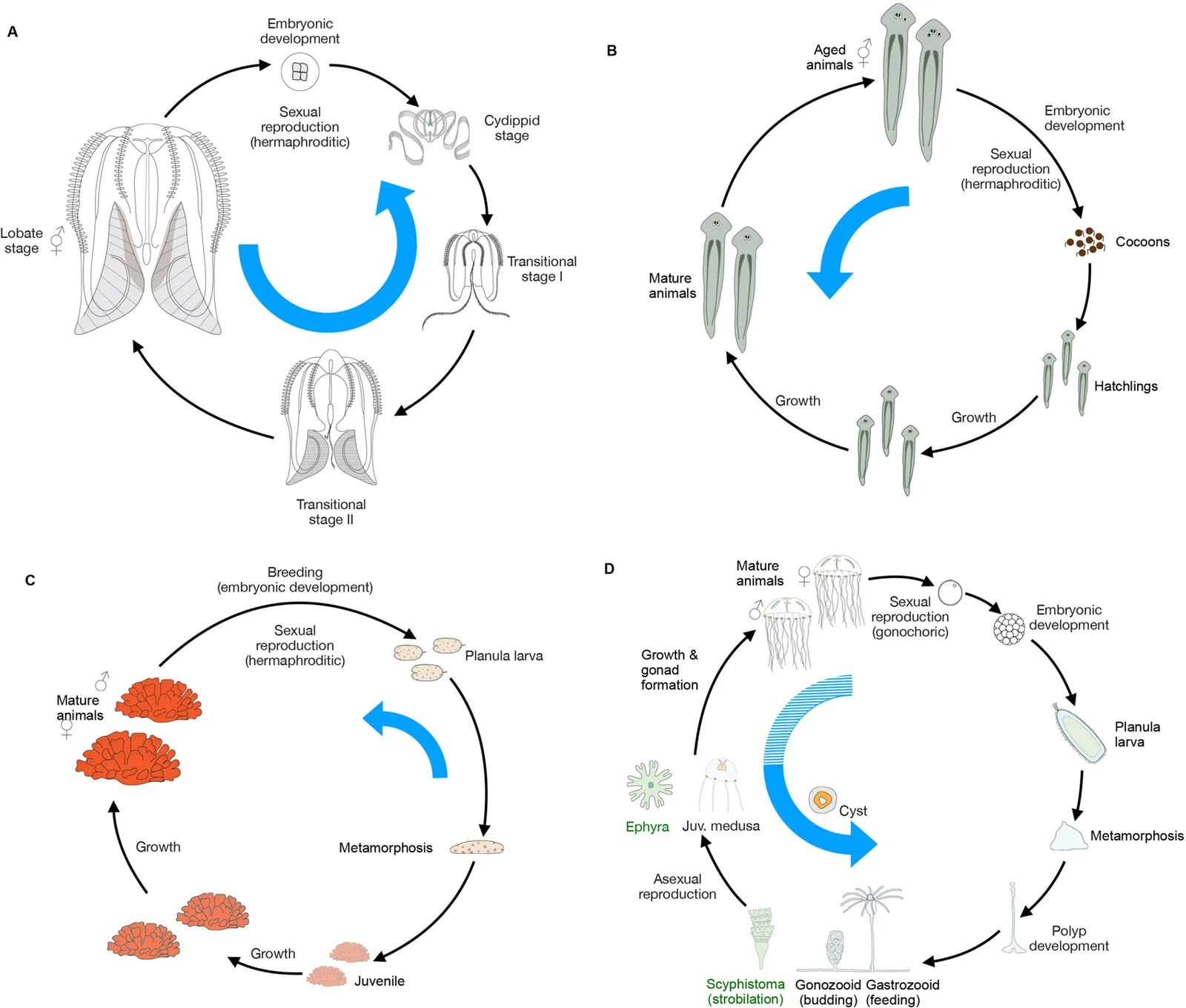
Diagrams illustrating simplified life cycles of four invertebrate phyla. (A) Ctenophora life cycle. Ctenophores exhibit a direct life cycle with external fertilization, in which eggs develop into cydippid larvae that gradually mature into adults without major metamorphosis. (B) Planaria life cycle. Planarians primarily reproduce asexually through fission, in which the body splits and each fragment regenerates into a complete individual, although some species can also reproduce sexually via cross-fertilization, leading to the formation of cocoons that hatch and develop into mature flatworms. (C) Cnidaria (Anthozoa) life cycle. Coral reproduction can be quite diverse even within a given species. Here, we report cross-fertilization-based sexual reproduction with internal breeding/development that leads to the release of a free-swimming planula larva. After settlement, the planula larva begins to metamorphose into a small coral colony that grows until sexual maturity. (D) Cnidaria (Medusozoa) life cycle. Cnidarians display a more complex cycle, often involving alternation between a sessile polyp stage and a free-swimming medusa stage; fertilization produces a planula larva that settles and metamorphoses into polyps, particularly the gonozooid, which later produces medusae by budding. A strobilating polyp (scyphistoma) and the resulting ephyra are depicted in light green. Note that the depicted life cycles are only examples of a relatively large variety of species-specific life cycles. This comparison highlights differences in reproductive strategies, larval forms, and developmental complexity among these groups. The blue arrows indicate reported morphologies of reversion/rejuvenation. The dashed blue part indicates that extended morphological reversion/rejuvenation periods are present in a subset of species.

## 3. Morphological reversion is widespread in aquatic invertebrates

Since the original observations of Van Beneden in 1844, who reported that the sea fur *Obelia* sp. medusae (Hydrozoa, Cnidaria) kept under laboratory conditions sporadically underwent MR to return to the polyp stage [26], scientists have observed that the usual developmental trajectories of a variety of aquatic metazoans are not always strictly followed. Most reports of MR triggered by various inductive stimuli at specific life stages have been extensively documented in cnidarians but also in ctenophores, platyhelminths and annelids (table 1, figure 2).

### (a) The well-documented case of morphological reversion in the immortal *Turritopsis dohrnii* jellyfish

One well-known cnidarian exemplifying MR induced by various stimuli is the hydrozoan *Turritopsis*, which gives it the famous name ‘*immortal jellyfish*’ (table 1)*.* Within this genus, MR has been particularly studied in *T. dohrnii*, whose sexually mature medusa can return to the polyp stage through an intermediate cyst stage multiple times in the same animal, without any noticeable limits [62]. This morphological phenomenon can be triggered by mechanical stress, starvation, temperature change, salinity variation or caesium exposure [20,29,30]. Spontaneously occurring MR has also been observed in ‘aged’ *T. dohrnii* medusae, suggesting that ageing may act as a trigger of this process and supporting the potential immortality of this specific species [29].

Interestingly, not only can the entire medusa undergo MR after induction but also parts of it can be isolated (e.g. the manubrium with cells from the exumbrellar epidermis) [29]. From a different cnidarian class, in the moon jellyfish *Aurelia aurita* (Scyphozoa), isolated tentacles from the scyphistoma (sessile, asexually reproducing polyps) launch MR by producing a motile planula-like stage that ultimately reforms a polyp [39]. Hence, MR from isolated structures has been observed in at least two distinct Cnidaria genera. Additional hydrozoans among *Turritopsis* species (e.g.* Turritopsis rubra*,* Turritopsis* sp.2,* Turritopsis* sp.5) have been described to undergo MR following mechanical stress (dissection, puncture) or starvation [33–37] (table 1). While MR has also been reported in the sexually mature medusa life stage in these species, the universality of this observation within this genus remains under debate [33,34,36–38].

### (b) Mechanical stress and starvation as triggers for morphological reversion

In several cnidarians, the mechanical detachment (using water turbulence, forceps compression, and scissor or needle injuries) of a budding immature medusa from its gonozoid (reproductive polyp) prior to its natural detachment can induce MR from the budding medusa back into a ball- or planula-like stage that first develops into a stolon and ultimately into a polyp (figure 2D). This has been described in different species, including the two hydrozoan jellyfish *Eleutheria dichotoma* [18] and *Cladonema pacificum* (previously *Cladonema uchidai*) [19], the smooth-spined snailfur *Podocoryna carnea* (previously *Hydractinia carnea*) [21] and *Podocoryna pruvoti* (previously *Hydractinia pruvoti*) [22]. This process, arrested at the stolon stage, was also proposed to occur in the hydrozoans *Perarella schneideri* (previously *Cytaeis schneideri*) and *Clytia hemisphaerica* [23].

In scyphozoans, mechanical stress (in the moon jellyfish *Aurelia* sp.1) [40] and starvation (in the thimble jellyfish *L. unguiculata* and in the crown jellyfish *Atorella* sp. and members of the *Nausithoe* genus; table 1) [20,29,40,41] have also been reported to induce MR, not only at the immature jellyfish ephyra stage, but also at the sexually mature medusa stage, by reverting these free-swimming life forms into polyps. While this latter observation is in line with reports from *Turritopsis* in which MR can be induced in sexually mature medusae, reports on the smooth-spined snailfur *P. carnea* indicate in contrast that the ‘maturity’ of the medusa bud inversely correlates with the rate of MR emergence following mechanical detachment [21]. These findings suggest a potential species-specific inhibitory role of sexual maturity in the MR process.

Interestingly, mechanical stress or starvation has also been reported to trigger MR outside of cnidarians. Their effects have been described in ctenophores, specifically in the sea walnut *Mnemiopsis leidyi* (table 1, figure 2A). After embryonic development, *M. leidyi*'s life cycle is characterized by two free-swimming, sexually mature stages: a bitentaculate cydippid stage, followed by a lobate stage in which tentacles are reabsorbed while lobes and auricles develop [65] (figure 2A). Recently, MR has been described in *M. leidyi*, showing that both mechanical stress (lobectomy) and prolonged starvation (followed by a low-food regimen) can induce reversal from the lobate stage to the cydippid stage, with fully functional tentacles [15]. It is interesting to mention that mechanical stress induced MR faster than starvation, and with a higher success rate: 40% (*n* = 15) versus 15% (*n* = 50), respectively [15].

Moreover, mechanical stress has also been described to induce MR in Platyhelminthes in the planarian freshwater flatworm *Schmidtea mediterranea* (table 1, figure 2B). A recent study on one of the sexual strains of *S. mediterranea*, which develops ectopic eye pigments with age, revealed that mechanical stress (amputation or injury) leads to the elimination of age-induced ectopic eye pigments and re-establishes a ‘younger’ phenotype (figure 2B). Interestingly, this is also observed when mechanical stress is applied without direct amputation of age-induced ectopic eye pigments [48]. In this study, a wealth of additional physiological and molecular analyses were performed, which are described in the rejuvenation section below.

### (c) Temperature as a trigger of morphological reversion

In addition to *T. dohrnii* (see above), temperature shifts have been described as inducers of MR, but thus far only in the following cnidarian species: the clapper hydroid *Sarsia tubulosa* (Hydrozoa), the hydrozoan jellyfish *Eucheilota paradoxica* (Hydrozoa), and the Mediterranean box jellyfish *Carybdea marsupialis* (Cubozoa). For *S. tubulosa*, slight temperature differences impact its life cycle [24]. At 2°C, *S.tubulosa* polyps bud medusae, whereas at 6–8°C, they do not. However, a shift in temperature from 2°C to 6–8°C induces premature detachment of the incomplete medusa. Depending on the stage of medusa maturity, these can undergo MR and revert to the polyp stage [24]. Similarly, in *E. paradoxica*, a temperature shift from 18°C (induction of medusa budding from the polyps) to 15°C induces MR of the newly formed medusa into a sessile polyp stage [25]. However, when the temperature is reduced from 18°C to 16–17°C, the medusa buds no longer develop into mature medusae but instead into small free-swimming, planula-like stages [25]. In *C. marsupialis*, the medusa is not budded but is directly formed by metamorphosis of the entire polyp, which can be triggered by a temperature shift from 20°C to 24°C. At early stages of metamorphosis, shifting the temperature from 24°C to 20°C induces MR*,* leading to the return of the organism to the polyp stage [42].

### (d) ‘Unfavourable conditions’ that trigger morphological reversion

In many published studies, the exact inducer of MR was not clearly identified or mentioned. The term ‘unfavourable conditions’ is used and encompasses environmental variations such as salinity, temperature, and/or food supply as inducers of MR from ephyrae back to the polyp stage in different scyphozoan species, such as the thimble jellyfish *Linuche unguiculata* and the crown jellyfish *Atorella* sp., *Nausithoe marginata*, *Nausithoe maculata*, *Nausithoe thieli*, *Nausithoe werneri*, *Nausithoe globifera* and *Nausithoe planulophora* [41]. Interestingly, ephyrae of the moon jellyfish *Aurelia* sp.1 have also been reported to undergo MR to the polyp stage under ‘overcrowded’ conditions [40]. In the hydrozoan *Laodicea undulata*, MR has been described to potentially occur under unfavourable conditions [27].

In the life cycle of another cnidarian, the cauliflower coral *Pocillopora damicornis* (Anthozoa), under natural conditions, when the free-swimming planula larva finds its corresponding cues (e.g. microbial biofilms), it metamorphoses into a sessile polyp on the ocean floor. It has been shown under laboratory conditions (exposure to a combination of environmental factors, including specific microbial films and light exposure at 24°C in stagnant water) that, up to 72 hours post-metamorphosis, the sessile polyp stage can undergo MR to become a planula-like motile stage [16]. Interestingly, upon returning to natural conditions, these induced planula-like corals can settle again and then metamorphose.

### (e) Morphological reversion in nonaquatic invertebrates

Occurrences of MR under unfavourable conditions (e.g. when leaked to other tissues or *in vitro* when conditions do not perfectly mimic the parasitic environment) have also been reported in the tapeworms *Echinococcus granulosus*,* Taenia serialis* and *Taenia multiceps* (Platyhelminthes)*.* During standard development, they undergo an indirect life cycle (figure 3A) that involves intermediate hosts (e.g. herbivores such as sheep) harbouring eggs, oncospheres and larval stages (metacestodes) or hydatid cysts (a specific metacestode unique to *E. granulosus*), as well as the protoscolex (juvenile infective scolex, i.e. head) formed by asexual budding of the cysts and a definitive host (e.g. carnivores such as dogs), where adults develop in the intestine [51]. When conditions are not optimal, the protoscolex stage, even as a strobilated and sexually mature adult (but only *in vitro*), has been observed to return to the hydatid cyst/metacestode stage [51].

**Figure 3.**
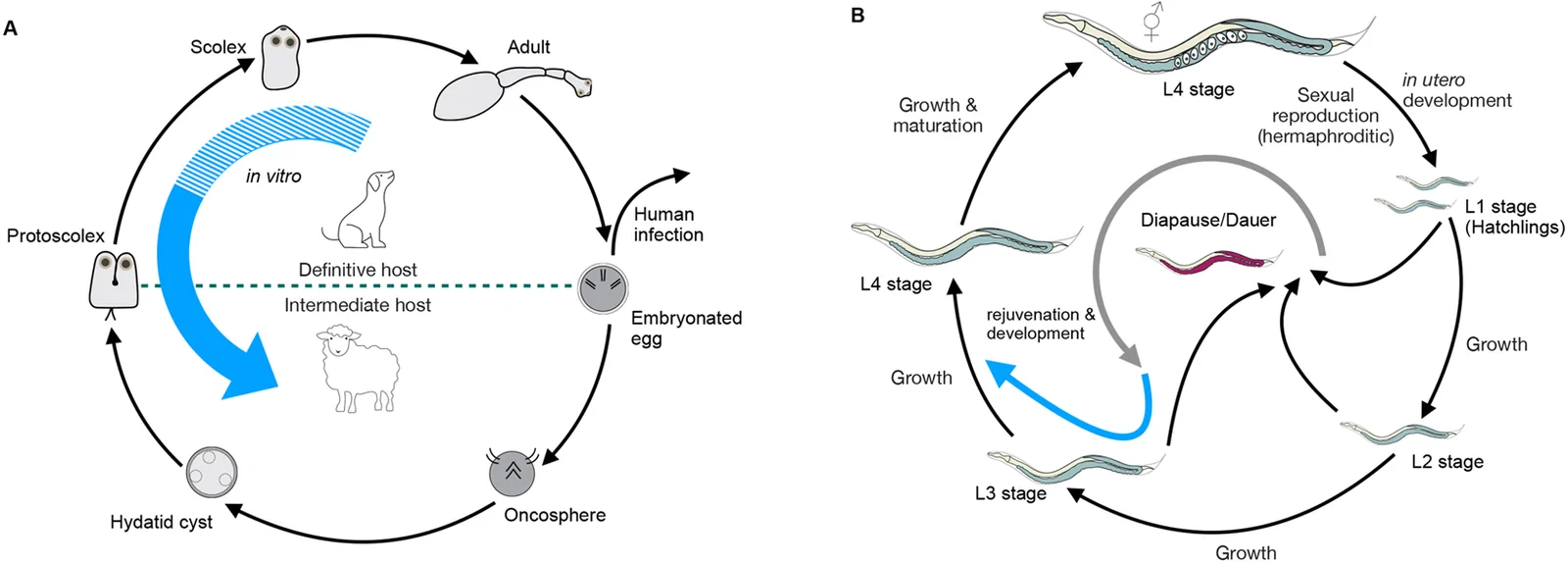
Diagrams illustrating simplified life cycles of two invertebrate phyla. (A) Parasitic Platyhelminthes life cycle. Parasitic platyhelminths, such as *Echinococcus granulosus*, typically exhibit a complex life cycle involving one or more hosts. Adults reproduce sexually within the definitive host, releasing eggs that develop into larval stages adapted for transmission. These larvae infect intermediate host(s), where they undergo further development or asexual multiplication before reaching the infective stage that returns to the definitive host and matures into an adult. (B) Nematoda life cycle. The nematode *C. elegans* reproduces sexually (primarily through self-fertilizing hermaphrodites), producing eggs that hatch into larvae progressing through four moults (L1–L4) before becoming adults; under unfavourable conditions, larvae may enter a Dauer stage specialized for survival and dispersal. As for figure 2, note that the depicted life cycles are only examples of a relatively large variety of species-specific life cycles. This comparison highlights differences in reproductive strategies, larval forms and developmental complexity among these groups. The blue arrows indicate reported morphologies of reversion/rejuvenation. The dashed blue part indicates that extended morphological reversion/rejuvenation periods are present in a subset of species.

## 4. Rejuvenation in aquatic invertebrates

While in all of the previously mentioned cnidarians, studies of the MR process have mostly focused on morphology, MR in the immortal jellyfish *T. dohrnii* has been characterized at multiple levels: morphological [29,31], cellular [31] and molecular [28,32]. Taken together, these studies support the idea that in this specific species, MR is possible through global tissue reorganization sustained by potential cellular transdifferentiation [29], as well as through the microscopic observation of degenerative and apoptotic processes [31]. Differential gene expression analysis was performed to compare the cyst, polyp and medusa stages. GO terms associated with ageing (e.g. determination of adult lifespan, multicellular organism ageing, ageing) decline globally in the cyst stage, whereas those associated with DNA synthesis, DNA repair, telomerase activity and telomere maintenance are enriched [32].

Furthermore, from an evolutionary perspective, a comparative study has been performed between the genomes of *T. dohrnii* and those of vertebrates and other cnidarian species, including the crimson jelly *T. rubra* [28]. *T. rubra* is presumably unable to undergo MR [28]; however, this remains under active debate [37,38]. Nonetheless, this analysis highlighted gene duplications (e.g. *xrcc5* and *rad51C*) in *T. dohrnii* that may potentially increase the efficiency of processes such as genomic instability regulation, telomere maintenance, epigenetic and transcriptional regulation, response to oxidative stress, cell cycle regulation, stem cell maintenance and regulation, and intercellular communication [28]. Moreover, unique protein variants have been identified for genes involved in some of these processes, further suggesting a higher efficiency of these mechanisms in *T. dohrnii* than in other cnidarians and vertebrates [28]. The perturbation of such processes associated with gene duplication in *T. dohrnii* is known to be linked to ageing in other organisms. Some have even been linked to lifespan extension in flies, nematodes and vertebrates [70]. Hence, the potentially increased gene expression associated with the duplicated genes might be a strong driver of rejuvenation in *T. dohrnii* [28,32]*.*

Since the early 20th century, rejuvenation has been a topic of interest in platyhelminth studies [17]. Child characterized the ‘*rejuvenescence*’ of different planarian freshwater flatworms, not at the morphological level but by assessing their metabolic rate, which is known to decrease with age, as in humans [17]. The metabolic rate was tested in freshwater flatworms using a debated susceptibility assay, i.e. the susceptibility of the organism to cyanide or potassium cyanide, which are metabolic poisons/cellular asphyxiants. With respect to the same dose, a younger individual (with a faster metabolism) dies faster than older individuals (with reduced metabolism) [17,71]. Using this assay, the metabolic rate was shown to increase after amputation in the freshwater flatworms *Girardia dorotocephala* (previously *Planaria dorotocephala*)*, Phagocata velata* (previously *Planaria velata*) and *Planaria maculata* compared to non-amputated individuals, restoring a ‘younger’ state [17]. In line with reports on the effects of starvation on MR induction (see above), starvation has also been reported to increase the metabolic rate in *G. dorotocephala* and *P. velata*, suggesting the induction of a ‘younger’ state [17,50].

In marine annelids, asexual reproduction was used as a physiological assay to determine the rejuvenation effects of mechanical stress and starvation. In the marine bristle worm *Parougia bermudensis* (previously *Dorvillea bermudensis*), the asexual reproduction rate of 6-month-old individuals is lower than that of newly regenerated individuals. Similarly to individuals resulting from regeneration, starving the 6-month-old *P. bermudensis* for 65 days leads to an increase in its asexual reproduction rate compared to continuously fed individuals [52]. Hence, in planarians and annelids, starvation and potentially asexual reproduction seem to increase metabolic and asexual reproduction rates, interpreted as a return to ‘younger’ stages by the authors of these studies and, ultimately, as a rejuvenation process.

To support this idea, it has been shown that mechanical stress (multiple amputations) in the marine flatworm *Macrostomum lignano* (Platyhelminthes) leads to potential rejuvenation, as assessed by examining the resulting individuals not only at the physiological level (i.e. mortality and fertility) but also at the morphological and molecular levels [43]. While fertility rates increased in the amputated animals compared with those in the controls (hinting at a potential rejuvenation), amputations were not shown to induce any decrease in the mortality rate, nor did durable morphological changes reverse age-associated morphology. Moreover, RNA-seq and subsequent PCA clustering analyses revealed that the main clustering factor was the age of the cohorts, with no or only minor effects of the amputations *per se*, indicating that potential rejuvenation could not be supported at the molecular level [43].

Similar investigations have been conducted in the freshwater flatworm *S. mediterranea*. Telomere length (TL), one of the hallmarks of ageing, has been shown to decrease with age for both sexually and asexually reproducing strains of *S. mediterranea*. A significant decrease in TL was observed one year post-fertilization for the sexual strain and 85–95 days post-fission for the asexual strain. Interestingly, in the asexual strain, three consecutive amputations led to an increase in TL in 85- to 195-day-old individuals, almost restoring the TL of 7-day-old individuals. However, in terms of sexual strain, amputations induce a greater decrease in TL in 6- to 12-month-old individuals, who display a TL similar to that of nonamputated 3-year-old individuals [49].

As described earlier in this review, the freshwater flatworm *S. mediterranea* displays ectopic eye pigments with age, which can be reverted following injury [48]. Hence, this amputation-mediated MR has been further investigated at the morphological, physiological, molecular and cellular levels [48]. Age-related changes in tissue composition, such as decreases in the levels of some neural cell types, increases in the number of glial cells in the head or the loss of body wall muscles, seem to be reversed by amputation. In one study [48], in contrast to what has been described in an earlier study [49], no similar decline in TL under physiological or amputation conditions was observed in the sexual strain, even in 20-year-old clonal colonies maintained only by amputation–regeneration cycles [48]. However, amputations restore (i) fertility, (ii) motility (which is reduced in older individuals) and (iii) free-radical and oxidative-stress levels to those of younger stages (by reduction). Furthermore, amputations induce tissue-specific reversal of age-associated transcriptional changes. High-throughput transcriptomic analyses (bulk- and sc-RNA sequencing) combined with correlation analysis of regenerated animals revealed that old amputated animals cluster with young ones and that certain, but not all, cell types (e.g. protonephridia) regain a younger molecular state [48].

## 5. Rejuvenation in the nematoda *Caenorhabditis elegans*

The life cycle of the nematode *C. elegans* starts with four different larval stages (L1, L2, L3 and L4; figure 3B) before it reaches adulthood [72]. In the case of unfavourable conditions, such as starvation, high population density or high temperature, *C. elegans* can enter diapause at different larval stages [73–76]. Diapause is a period of arrested development in crustaceans, insects and fish, as well as in nematodes [77]. Here, while diapause extends the overall survival period of *C. elegans*, we consider its lifespan only as the period that does not include any diapause arrest. Hence, during diapause, the survival of individuals is extended, and they are able to re-enter normal life cycles under favourable conditions [54,55,73]. Moreover, the lifespan of recovered individuals is not affected by the duration of diapause [54–56]. This led to the hypothesis that ageing was paused during diapause [54,55].

However, mutations known to increase or decrease the lifespan of *C. elegans* have been shown to affect the survival of arrested L1 larvae. The latter experience cellular decline similar to that of normal ageing, but this can be reversed by feeding [57]. Furthermore, different studies have shown that wild-type specimens entering diapause still present increasing ageing hallmarks over time. In fact, when they enter diapause from the L1, L2 or L3 stage (L3 is also called dauer; [73]), they acquire a granular appearance, increased mortality, increased sensitivity to heat and increased accumulation of ROS and aggregated proteins. In addition, these resulting dauer larvae exhibit decreased oxygen consumption and mitochondrial performance, as well as transcriptional changes similar to those described in old adult *C. elegans* [56,58,59,78–85].

Interestingly, upon recovery from diapause, many of these changes are partially reversed [58]. While certain ageing hallmarks, such as protein aggregation, are not reversed after recovery [56], animals that have recovered from L1 arrest present restored viability, increased motility, low ROS levels, the replacement of fragmented mitochondria by tubular ones, and restored mitochondrial DNA quantity [56,60,61]. The accumulation of lysosomal damage caused by macroautophagy during L1 arrest is reversed after recovery via the unfolded protein response gene *ire-1* [56,86]*.* Similarly, after the recovery of animals arising from the L3 stage, oxidized flavins are not reversed; however, oxygen consumption, heat production, ATP concentration and mitochondrial activity increase again, as in younger animals [58]. Upon dauer exit, in addition to a recovery from transcription-blocking DNA damage, a reversal of age-associated transcriptional changes occurs [59].

Hence, combining several data sets strongly supports the conclusion that rejuvenation occurs in nematodes, particularly in *C. elegans*, where it is observed in animals exiting diapause from L1 and/or L3. In fact, when favourable conditions return, the nematode exits diapause and resumes normal development, showing signs of restored/rejuvenated cellular function.

## 6. Mechanical stress as a means to extend lifespan?

The physiological rejuvenation effects of amputation in the freshwater flatworm *P. maculata* (Platyhelminthes) have led to the idea that repeated amputation might also extend its lifespan, i.e. maintain the same individual indefinitely through repeated rejuvenation inductions. To test this idea, *P. maculata* was amputated 16 times over a period of one and a half years. However, this experiment ended because almost all of the individuals died between the 13th and 16th amputations because of external factors [17]. Nevertheless, multiple sequential amputation experiments have also been conducted in other organisms. For example, in the aquatic oligochaete worm *Paranais litoralis* (Annelida), three sequential amputation–regeneration cycles were proposed to increase the lifespan of the animal from around 65 days in nonamputated controls to around 75 days. However, because lifespan in nonamputated controls seems to vary between around 65 and 80 days [53], additional experiments are needed to confirm the robustness of these results.

In line with these observations, the lifespan of another platyhelminth species, the marine flatworm *M. lignano*, has been argued to have extended from 10 months in the control group to 12 months following twenty-nine successive amputations [44]. Moreover, this experiment has been continued for more than two years, reaching fifty-nine successive amputations [45,46]. While these results seemed accepted at first [45,46], they have since been debated because of the apparent short lifespan of the control group. Indeed, *M. lignano* was shown to survive for close to two years (745 days) under laboratory conditions, questioning the previously described lifespan extension effect of the amputations [47]. The relatively short lifespan of Egger's control group [44] may have been caused by pathogen contamination that may have been eliminated by the amputations, thus explaining the observed lifespan extension [47].

## 7. Discussion

### (a) Pan-metazoan occurrences of morphological reversion/rejuvenation

In addition to experimentally induced reprogramming and rejuvenation in mammals (e.g. parabiosis, 4F reprogramming), stimuli or spontaneously occurring MR/rejuvenation are present in various invertebrate phyla, most of which are marine/aquatic. To what extent MR/rejuvenation has a common evolutionary origin or is the result of convergent evolution remains largely unknown and requires the study of a larger number of metazoan species (table 1). The phylum with the most described occurrences of MR/rejuvenation is Cnidaria, potentially because of the ease of determining the morphological changes associated with their life cycles. Among the various stimuli, mechanical stress and/or starvation appear to be the major pan-metazoan triggers of MR/rejuvenation. In fact, both stimuli have been shown to trigger this process in Ctenophora, Cnidaria, Platyhelminthes, and Annelida (table 1). These observations from diverse invertebrate species and phyla suggest potential evolutionarily conserved mechanisms triggering this process. What consequences these stimuli have at the molecular level remains an open question and will provide crucial insight into the evolutionary trajectories of MR/rejuvenation in metazoans.

### (b) Stress resistance versus morphological reversion/rejuvenation

MR/rejuvenation-sensitive life stages in certain species seem to be restricted to specific stages (e.g.* T. dohrnii*,* C. elegans*, table 1). These stages (e.g. early medusae in hydrozoan cnidarians) might be more sensitive to environmental variations than polyps are, leading to a restrictive permissive window; the MR/rejuvenation process could therefore be a stress-resistance strategy rather than one aimed at lifespan extension. Unfortunately, systematic assessments of the effects of environmental conditions on polyps and medusae of the same species are nonexistent or sparse. One study reported the strong resistance of polyps from the Asian moon jellyfish *Aurelia coerulea* to food scarcity and low temperatures in their natural habitat (no MR/rejuvenation reported for this species; [87]). On the other hand, the ephyrae of the upside-down jellyfish *Cassiopeia xamachana* appear sensitive to food availability, an environmental condition from which it can nonetheless recover (no MR/rejuvenation reported for this species; [88]). While it remains unclear whether the polyp stage from a given species is more resistant than its corresponding medusae stage, these data are in line with the idea that MR/rejuvenation activates molecular stress-resistance mechanisms that lead to cellular reprogramming and a return to a more resistant life stage. If this is the case, one could reconsider the sporadic, age-induced MR observed in *T. dohrnii* as arising from the accumulation of cellular damage and functioning as an induced stress response that subsequently leads to rejuvenation.

### (c) Regeneration, asexual reproduction and rejuvenation

Interestingly, a large number of cnidarians, platyhelminths and annelids are characterized by remarkable regenerative capacities, in addition to their ability to reproduce asexually [89]. The relationship between regeneration and asexual reproduction is an active field of research, highlighting similarities and process-specific features. This is especially relevant because asexual reproduction in these organisms may involve the reformation of missing structures [90,91]. Independent of the precise relationship between these two developmental trajectories, the persistence of asexual reproduction across evolutionary timescales suggests that it is linked to survival at the population level [92].

Here, we distinguish between individual lifespan and clonal lifespan. Throughout this review, the term lifespan refers to individual lifespan. The continuous maintenance of a population through asexual reproduction over an indefinite period implies a potentially infinite clonal lifespan, but not necessarily an infinite lifespan for each individual organism. Evidence from asexual reproduction in yeast indicates that the process can be asymmetric, with one daughter cell inheriting ageing-associated damage while the other undergoes a form of rejuvenation with a reset of the ageing state compared to the mother cell [93]. Whether analogous mechanisms operate at the organismal level in asexually reproducing metazoans remains unknown. Consequently, two scenarios may explain how an effectively infinite clonal lifespan can be achieved: (i) the animals of a given species exhibit inherently negligible organismal senescence, or, more probably, (ii) asexual reproduction involves a reset of ageing hallmarks in at least one of the resulting organisms.

Considering the latter, if regenerative or wound-healing processes fully or partially mirror the mechanisms that underlie asexual reproduction, then mechanical-stress-induced rejuvenation may simply be a secondary consequence of the rejuvenation programmes already inherent in asexual reproduction. These findings suggest that regeneration and starvation trigger MR/rejuvenation responses through distinct mechanisms. Overall, determining process-specific (i.e. regeneration versus asexual reproduction) or shared mechanisms associated with rejuvenation remains largely unexplored and warrants systematic comparative investigations.

In addition to the injury-induced rejuvenation described in planarians (e.g. restoration of young eyes [48]), only a few studies in vertebrates are beginning to suggest a link between regeneration and rejuvenation. In fact, it has been proposed that the redeployment of embryonic molecular programmes during regeneration (e.g. in amphibians) could be associated with a transient youthful state [94]. How this state is maintained over the long term in regenerated tissue or whether the new tissue is of equivalent age as the initially developed tissue [95] remains unknown. In line with this idea, the observation that regenerated skin in axolotls appears thicker and presents increased dermal connective tissue [95] suggests rejuvenation of this tissue [96]. The in-depth construction of DNA methylation clocks to predict early life periods in axolotls [97] enables, as suggested by the authors, ‘explorations into how axolotl regeneration affects epigenetic age’. Following up on these observations would be crucial to determine the potential rejuvenating effect of regeneration, the underlying molecular mechanisms, and its evolutionary conservation from invertebrates to vertebrates.

### (d) Rejuvenation and lifespan and/or healthspan extension

The absence or presence of detectable rejuvenation across levels in only a subset of the assessed ageing hallmarks (e.g. fertility, TL, stem cell content and mitochondrial activity [70]) may reflect an incomplete rejuvenation process. Alternatively, this may further reflect the need to reconsider the relevance of these hallmarks to the ageing process in these nonconventional, long-lived animals and, consequently, their relevance to assessing rejuvenation. In addition, ageing hallmarks are highly interconnected, with some contributing more critically than others to organismal ageing and, potentially, to rejuvenation [70]. This raises a conceptual question about the use of the classical hallmarks of ageing, which were characterized primarily in conventional model organisms and whose conservation across metazoans has already been questioned [70,98]. More globally, this further highlights the complexity of assessing the effects of rejuvenation on these nonconventional, long-lived organisms and the need for additional criteria.

From a functional perspective, complete or partial rejuvenation should lead to the extension of an organism's lifespan. This lifespan extension could be further enhanced through successive stimuli, allowing one to distinguish the long-term outcomes of partial versus complete rejuvenation. If rejuvenation is partial (i.e. only certain aspects of physiology or certain cell types are affected), successive inductions can still result in the gradual ageing of the organism, ultimately leading to death. This may have been the case for *P. maculata*, were it not for external cues as indicated by Child [17]. In contrast, if rejuvenation is ‘complete’ at all levels, successive inductions should enable the animal to escape the ageing process, leading, under extreme conditions, to biological ‘immortality’, such as in the immortal jellyfish *T. dohrnii* [28,62]. While rejuvenation may extend the lifespan of certain invertebrates, this question remains largely unresolved and presents challenges, especially in those species for which lifespan is unknown or already naturally extended.

In this regard, the assessment of repeated amputations and their effects on rejuvenation/lifespan extensions in the freshwater flatworm *S. mediterranea* and the marine flatworm *M. lignano* will further highlight this point. The ability of an *S. mediterranea* population to be maintained solely by amputation–regeneration cycles for 20 years is impressive [48]. However, it remains unclear whether amputation, which induces rejuvenation events at multiple levels in this species [48], also extends its lifespan. This is mainly because of the absence of an experimentally determined maximum lifespan for *S. mediterranea.* Hence, whether rejuvenation has no effect on an animal's lifespan or whether *S. mediterranea* can escape ageing under physiological conditions remain unknown, making this question less relevant. Mechanical stress in *M. lignano* increases fertility rates to those of younger stages but has no global rejuvenation effect at the morphological level (only temporary changes) or at the molecular or mortality/lifespan levels [43]. However, the absence of apparent effects of amputation on the absolute lifespan might simply be because of the length of the experiment, which did not exceed *M. lignano*'s lifespan [43].

Healthspan, a broadly used term referring to the period of good health and performance [99] and intrinsic capacity, defined by the World Health Organization as ‘the composite of all the physical and mental capacities that an individual can draw on at any point in time’ [100], are more recent concepts that could also be taken into account when assessing the effects of rejuvenation on a given organism. Although these two concepts somewhat overlap, intrinsic capacity can be considered the organism's overall functional state at a given time (i.e. multitrait score), whereas healthspan refers to the amount of time an organism lives in a healthy, functional state (i.e. the duration during which this multitrait score remains above a certain threshold). Hence, using intrinsic capacity and healthspan, i.e. the combination of motor or sensory function, tissue integrity, physiological reserve and reproductive function at a given time or over a period of time to group various physiological and behavioral readouts, could help formalize whether rejuvenation improves organismal performance.

While the definitions of intrinsic capacity and healthspan remain inconsistent [99], both require multidimensional assessments (physiology, behaviour, etc.) and thus far have, *sensu stricto*, not been used in long-living (or rejuvenated) invertebrates. Specifically, locomotion or responses to light can be used as indicators of neuromuscular function [48,101]; quantifying muscle or neuron content may reflect metabolic state [102] or tissue integrity; assessing egg production and fertility in sexually reproducing animals or bud formation in asexual animals can inform reproductive capacity; and evaluating regeneration after single or repeated injuries under normal or stressed conditions can be used to assess functional restoration. Overall, using the concepts of intrinsic capacity and healthspan could provide meaningful insight and complementary information on the molecular effects of rejuvenation in the absence of a determinable effect on lifespan extension.

### (e) A consensual approach to determine rejuvenation in invertebrates

On the basis of these considerations, we propose the following integrative strategy to investigate the occurrence of rejuvenation in nonconventional metazoan research models. First, the baseline ageing trajectory of the organism should be characterized via the identification of relevant multidimensional ageing hallmarks at the morphological, physiological (e.g. fertility decline, reduced healthspan/intrinsic capacity, increased mortality), cellular (stem cell exhaustion, cellular senescence, etc.) and molecular (transcriptome, methylome, TL, etc.) levels and, ideally, the determination of its lifespan. At a minimum, and particularly for species with long lifespans where complete lifespan studies may be impractical, organisms should be monitored over a sufficient period to observe the emergence of age-associated phenotypes. In this context, observable morphological and physiological deterioration may provide an initial indication of ageing, although more detailed analyses are recommended.

These elements (ageing hallmarks and lifespan) can then be used to assess the effects of spontaneous or stimuli-induced MR to determine whether rejuvenation has occurred. Furthermore, the effects of both single and repeated inductions should be assessed as potential triggers or amplifiers of rejuvenation. Finally, if applicable, lifespan extension can be addressed over a representative time frame (i.e. at least exceeding the organism's initial lifespan).

### (f) Leveraging the potential of rejuvenation in invertebrates to fight ageing in mammals

Comparative ageing biology is an active and growing field. Various ageing-related hallmarks have been identified [103], and as ageing research has advanced, additional hallmarks have been identified [70,104]. Among the various therapeutic approaches aimed at counteracting the deleterious effects of ageing and its associated hallmarks, cell and tissue rejuvenation is of crucial interest [105,106]. Some of these approaches attempt to rejuvenate cells, tissues and organs using partial 4F reprogramming [107] or insights obtained from heterochronic parabiosis [6]. While these approaches are very promising, they still face significant experimental and regulatory challenges associated with the unknown long-term consequences of epigenetic reprogramming in humans (e.g. genomic instability and tumourigenicity) [106].

This is where we consider the spontaneous (i.e. ageing-associated) or stimulus-induced rejuvenation observed in invertebrates as a complementary opportunity for ageing research. In fact, the above-described rejuvenation occurrences (table 1) appear not to be associated with any visible signs of pathology or aberrant outgrowth (i.e. hyperplasia), suggesting that the underlying mechanisms involve a coordinated and balanced molecular network.

Yamanaka factors (OSKM: Oct4, Sox2, Klf4 and c-Myc; [7]) or their slightly modified Thomson factors that reduce the tumourigenic effects of c-Myc (OSNL: Oct4, Sox2, Nanog, Lin28; [108]) have been widely studied for cellular reprogramming and rejuvenation, mainly in vertebrates, especially mice and humans [8–11]. Nanog has been described as a vertebrate-specific innovation [109]. However, a recent study suggested that a vent-like transcription factor could mimic ancestral Nanog activity, as it is capable of reprogramming pluripotency in invertebrate deuterostomes [110]. This study further suggested that vent-like factors from more early-branching metazoans, such as cnidarians, may have retained a partial reprogramming capacity [110], highlighting that previously unsuspected invertebrate transcription factors could be involved in these processes.

Initial reports have suggested that only Sox2 and c-Myc, together with gene families corresponding to other factors, might be conserved in early-branching metazoans [111,112]. However, their precise orthologies are less definitive [112,113]. A recent study has assessed, among other genes (i.e. Sirtuins, HSPs and telomere-associated genes), the potential Yamanaka/Thomson factors conserved in *T. dohrnii* during the rejuvenation process, revealing the upregulation of Sox2-like, Klf-like and Pou-like genes when reverting from the cyst stage to the polyp stage [113]. The precise orthology of these factors to the Yamanaka/Thomson factors, as well as their roles in rejuvenation/reprogramming in *T. dohrnii*, in which transdifferentiation has been proposed to be involved [29], remains to be determined.

Nonetheless, as in Matsumoto & Miglietta [113], these reprogramming factors can serve as candidate genes to determine their expression activity during rejuvenation in invertebrates. In addition, bulk RNA-seq and especially scRNA-seq data could provide unbiased insights into additional mechanisms and reveal additional factors involved in reprogramming in these animals. A particular emphasis should be placed on comparing the molecular programme to the embryonic developmental programme, as this comparison could provide additional insight into shared as well as rejuvenation-specific factors/mechanisms. Combined with the global genomic conservation observed within metazoans [114,115], especially between cnidarians and vertebrates [116], these findings may lead to the identification of factors/molecular networks involved in reprogramming during organismal rejuvenation that could be transferred to mammalian models.

## 8. Conclusion and perspectives

Naturally occurring rejuvenation events have been reported from different phyla across the metazoan tree of life. While many experimental approaches to rejuvenating vertebrates through parabiosis or cell reprogramming are being extensively investigated, the mechanisms underlying spontaneous or stimulus-induced rejuvenation in invertebrate metazoans remain in their infancy and are still largely descriptive. However, the current literature highlights an impressive number of rejuvenation-like processes in a variety of evolutionarily distant animals that can be triggered by shared stimuli. Taking into account the heterogeneity in terms and approaches used to describe the occurrence of this process in invertebrates, we consider it important (i) to distinguish between MR and rejuvenation, (ii) to use multilevel approaches to determine the precise effects of rejuvenation on the organism, and (iii) to determine the effects of rejuvenation on the lifespan of the animal, if not in an animal exhibiting negligible senescence. In the latter, assessing healthspan/intrinsic capacity should be considered. Finally, (iv) molecular assessments of this process should include candidate genes (e.g. Yamanaka/Thomson factors) as well as unbiased and comparative (e.g. development versus rejuvenation) approaches to determine the factors/molecular networks involved in reprogramming during organismal rejuvenation. Thus, describing and studying MR/rejuvenation using a systematic strategy that considers developmental programmes for intra- and interspecies comparative studies is a critical point that could also represent novel opportunities for the development of innovative anti-ageing therapies.

## Data Availability

This article has no additional data.

## Theme

One contribution of 21 to a theme issue ‘Developmental circuits in ageing’.
